# Routine COVID-19 testing may not be necessary for most cancer patients

**DOI:** 10.1038/s41598-021-02692-3

**Published:** 2021-12-02

**Authors:** Ali Motlagh, Fatemeh Elmi, Maisa Yamrali, Mansour Ranjbar, Mehrdad Azmin, Farzaneh Moshiri, Christoph Hamelmann, Slim Slama, Nadia Tavakoli, Asmus Hammerich, Nasim Pourghazian, Marzeyeh Soleymani Nejad, Ahmad Mafi, Payam Azadeh, Maryam Aghajanizadeh, Afshin Ostovar, Alireza Raeisi, Reza Malekzadeh

**Affiliations:** 1 Department of Radiation Oncology, Imam Hossein Hospital, Shaheed Beheshti Medical University, Tehran, Iran; 2 Cancer Research Center, Shaheed Beheshti Medical University, Tehran, Iran; 3 National Cancer Control Secretariat, Ministry of Health and Medical Education, Tehran, Iran; 4 WHO Country Office, Tehran, Iran; 5 Non-Communicable Diseases Research Center, Endocrinology and Metabolism Population Sciences Institute, Tehran University of Medical Sciences, Tehran, Iran; 6 Department of Biotechnology, School of Medicine, Alborz University of Medical Sciences, Karaj, Iran; 7 WHO Regional Office for Eastern Mediterranean Region, Cairo, Egypt; 8 Department of Cell and Molecular Sciences, Faculty of Biological Sciences, Kharazmi University, Tehran, Iran; 9 Osteoporosis Research Center, Endocrinology and Metabolism Clinical Sciences Institute, Tehran University of Medical Sciences, Tehran, Iran; 10 Department of Internal Medicine, Shiraz University of Medical Sciences, Shiraz, Iran; 11 Deputy of Public Health, Ministry of Health and Medical Education, Tehran, Iran; 12 Digestive Disease Research Institute, Tehran University of Medical Sciences, Tehran, Iran

**Keywords:** Cancer, Cancer, Infectious diseases

## Abstract

Cancer patients are at risk for severe complications or death from COVID-19 infection. Therefore, the need for routine COVID-19 testing in this population was evaluated. Between 1st August and 30th October 2020, 150 cancer patients were included. Symptoms of COVID-19 infection were evaluated. All eligible individuals went through RT-PCR and serological tests for COVID-19. At the same time, 920 non-cancer patients were recruited from a random sample of individuals who were subject to routine molecular and anti-body screening tests. Of 150 cancer patients, 7 (4.7%) were RT-PCR positive. Comorbidity made a significant difference in the RT-PCR positivity of cancer patients, 71.4% positive versus 25.8% negative (*P*-value = 0.02). The average age for negative and positive groups was 53.3 and 58.2 respectively (*P*-value = 0.01). No significant difference was observed between cancer and non-cancer patients regarding COVID-19 antibody tests. However, cancer patients were 3 times less likely to have a positive RT-PCR test result OR = 0.33 (CI: 0.15–0.73). The probability of cancer patients having a positive routine test was significantly lower than non-cancer patients, and the concept that all cancer patients should be routinely tested for COVID-19 may be incorrect. Nevertheless, there may be a subgroup of patients with comorbidities or older age who may benefit from routine COVID-19 testing. Importantly, these results could not be subjected to multivariate analysis.

## Introduction

Coronavirus disease 2019 (COVID-19), the new disease caused by the novel coronavirus (severe acute respiratory syndrome coronavirus 2 (SARS-CoV-2)), was declared a pandemic on March 11, 2020^1^. Many countries throughout the world are struggling with a disease that poses a significant risk of severe complications or death to older individuals (more than 60 years old), and ones with comorbidities or cancer^2^. Patients with cancer are specifically at risk for adverse outcomes from COVID-19 infection as their immune system is compromised due to the cancer itself or the treatment they receive (including surgery, chemotherapy, immunotherapy or radiotherapy)^3^.

According to one of the earliest studies in China, of 1590 COVID-19 cases analyzed, 18 patients had a history of cancer. Patients with cancer were older and had more severe baseline computed tomography (CT) manifestations compared to others and more importantly they had a higher risk of severe events (admission to the intensive care unit, requiring invasive ventilation, or death)^3^. According to the World Health Organization (WHO) COVID-19 dashboard, the overall mortality rate of this disease is about 2% in the general population, as of September 2021^4^. However, the mortality rate in cancer patients has been reported to be higher than the general population reaching to 25.6%^5^.

One of the first reports describing the characteristics of COVID-19 infection in cancer patients reviewed 1524 cancer patients and found 12 patients infected with SARS-CoV-2, only 5 of them receiving treatment at the time of contracting the infection. They concluded that the risk of COVID-19 infection in cancer patients was about 2 times than that of the general population^6^.

A retrospective study by Zhang et al. evaluated the clinical characteristics of 28 cancer patients infected with SARS-CoV-2. In this study severe complications happened in 53.6% (15 patients) and the mortality rate was 28.6%. Interestingly, they reported that receiving anti-tumor treatment within 14 days of COVID-19 diagnosis was associated with an increased risk of developing severe events^7^.

Therefore, during the treatment process in these patients, some measures need to be taken, such as postponing elective surgeries in cancer patients in endemic areas, implementing more personal protective regulations for them, and providing more intensive care and treatment in patients infected with this virus.

Deciding whether to approve a scheduled prescription in cancer patients or delay treatment depends on the biological characteristics of the tumor, the patient's clinical condition and symptoms, therapeutic characteristics (expected benefits or side effects including myeloid suppression), disease response, current anti-cancer treatment and potential risk of coronavirus.

On the other hand, the health system and treating physicians may face a great challenge differentiating clinical symptoms of COVID-19 in patients with cancer. Cancer patients may have symptoms mimicking COVID-19 especially in cases with lung cancer or lung metastases who present with cough or dyspnea, and the diagnosis of this infection based solely on the clinical presentations would be difficult^8^. Furthermore, due to their clinical condition and suppressed immune system, they may not have the typical symptoms of COVID-19 infection^9^. Therefore, this group of patients or a subgroup of them (including a specific age group, specific types of cancers, or being in a particular stage of the disease or receiving specific treatment) may need to undergo non-clinical evaluations routinely, regardless of suspected symptoms.

There are some reports describing the characteristics and outcomes of COVID-19 in cancer patients^10,11^, but to our knowledge no such study has been conducted in our country. Due to the current urgent situation in the country and the limited information about COVID-19 in cancer patients, the need for a study regarding the epidemiology and characteristics of COVID-19 disease in cancer patients in our country is felt more than ever.

The aim of this study was to evaluate the clinical and laboratory characteristics of COVID-19 disease in cancer patients and determine whether routine COVID-19 testing is necessary in this population.

## Methods

### Patients

#### Cancer patients

The present study is a retrospective case control study on 150 cancer patients from all over Iran who were referred to be treated for cancer in the clinical oncology department of Imam Hossein Hospital, a tertiary cancer center, in Tehran from 1st August to 30th October of 2020. The ethical approval required for this study was received from the Deputy for Research and Technology of Shahid Beheshti University of Medical Sciences, registration number: IR.SBMU.RETECH.REC.1399.476. Date: 22 Sep 2020. All methods were performed in accordance with the guidelines and regulations of the mentioned institution.

Cancer patients were considered eligible for this study whether they were on active anti-cancer treatment or not (follow-up patients), and regardless of the treatment modality employed (systemic therapies, radiotherapy, surgery). Minimum enrollment age was 20 years. Patients were evaluated for common symptoms of COVID-19 infection and reverse transcription polymerase chain reaction (RT-PCR) test along with serological test for COVID-19 were performed for all eligible individuals at the beginning of the study. All patients signed a consent form, and were fully informed about the interventions. The patients were given the choice of not undergoing one of the tests (RT-PCR or serology) if they were not willing to. Nevertheless, it did not result in omitting them from the study.

Special data collecting forms were designed for cancer patients. Patient-related information (personal information) and records of previous and underlying diseases (diabetes, cardiovascular disease, hypertension, chronic lung disease), cancer characteristics (including type and stage), and cancer treatment types (surgery, systemic treatments and radiotherapy) were recorded. The form contained a section where the results of the initial RT-PCR and serology test were recorded.

If any of the tests were positive, the patient was referred to the Corona Care Center and the results of further examinations for clinical symptoms (asymptomatic, fever, dry cough, shortness of breath, etc.), laboratory findings (CBC, serum albumin, LDH, CRP, ESR, D-Dimer), imaging findings, hospitalization conditions (ICU, non-ICU), treatments for the disease, and treatment outcome were recorded. These patients were monitored weekly regarding their general condition and treatment progress.

#### Control groups

To allow comparison with a control group, non-cancer patients were recruited from a random sample of individuals who were subject to routine molecular and anti-body screening tests. The testing period of time was the same as cancer patients (August to October). Minimum recruitment age for this group was 20 years. The age distribution in the molecular test group comprised of 61.6% 20–39, 34% 40–64, and 4.3% 65 and above. The control group consisted of individuals working in companies who requested their employees and their families to undergo COVID-19 testing as a screening test. The tests were performed in the same laboratory as for cancer patients.

### Laboratory tests

The COVID-19 diagnostic survey was performed through nucleic acid amplification test (NAAT) RT-PCR test to assess acute infection and enzyme-linked immunosorbent assay (ELISA) for immunoglobulin G (IgG) and immunoglobulin M (IgM) to provide evidence of prior infection with SARS-CoV-2.

For RT-PCR assay, nasopharyngeal and oropharyngeal swabs were collected from asymptomatic individuals. Swabs were placed in viral transport media (VTM) and transferred to the laboratory on the day of collection. The viral RNA was extracted by QIAamp 96 Virus QIAcube HT Kit using QIAcube HT automated nucleic acid purification (Qiagen, Hilden, Germany) according to the manufacturer instruction. The multiplex one-step RT-PCR was performed using primers and taqman probes for conserved region of N and OrF1ab gene of SARS-CoV-2 and RNase P as internal control. The amplification conditions were 50 °C for 30 min, 95 °C for 1 min followed by 45 cycles of 95 °C for 15 s and 60 °C for 30 s. Real time RT-PCR was run in Rotor Gene 6000 cycler (Qiagen, Hilden, Germany).

Enzyme immunoassay for the qualitative determination of the presence of anti-SARS-CoV-2 IgG and IgM was performed in fresh plasma. ELISA used specific SARS-CoV-2 nucleocapsid antigens and was done according to the manufacturer instruction (Pishtaz teb co., Iran).

### Statistical analysis

Fisher’s exact test was conducted to test the independence of demographic and clinical characteristics of cancer patients and laboratory test results. Fisher’s exact test was conducted between cancer and control groups to test the independence of having cancer from laboratory test outcomes. Difference in age was tested using Welch’s T-test. Statistical analysis was performed using Python 3.7 and statistical tests were conducted using SciPy package. A P-value of less than 0.05 was considered as statistically significant. Since the number of main events in our study was less than 10, we could not do a multivariate analysis for various clinical characteristics.

## Results

### Participants

In the period between 1st August and 30th October of 2020, 150 patients with cancer were enrolled. Median age for this group was 53 (IQR = 17) with 21.3% for 20–39, 63.3% for 40–64, and 15.4% for 65 and above. For the control groups, 129 participants from the anti-body group met the inclusion criteria. The molecular test group comprised 1353 individuals, 920 of whom met the inclusion criteria and entered the study. The age distribution in the molecular test group comprised of 61.6% for 20–39, 34% for 40–64, and 4.3% for 65 and above with median of 37 years (IQR = 14).

### Test results

We performed three different COVID-19 molecular and antigen tests for each patient. The test results and clinical description of the patients are illustrated in Table 1.

**Table 1:** Characteristics and outcome of 150 cancer patients tested for COVID-19.

	RT-PCR result			IgM serology result			IgG serology result		
	Pos	Neg	*P*-value	Pos	Neg	*P*-value	Pos	Neg	*P*-value
**Age**									
Mean (std)	59.7 (5.5)	52.5 (12.8)	**0.01**	51.0 (11.6)	53.9 (12.8)	0.61	55.1 (8.6)	53.5 (13.3)	0.55
**Sex**									
Female	4	88	1.0	3	65	1.0	11	57	0.56
Male	3	55		2	37		4	35	
**Comorbidity**									
None	2	106	**0.02**	3	69	0.66	7	65	0.08
At least one	5	37		2	33		8	27	
**Stage**									
1 to 2	2	61	0.70	3	43	0.65	7	39	0.77
3 to 4	4	79		2	56		7	51	
**Cancer journey state**									
Treatment	4	113	0.18	3	83	0.25	12	74	1.0
Follow-up	3	30		2	19		3	18	
**Type of treatment**									
Chemotherapy	7	103	0.19	4	77	1.0	13	68	0.18
Radiotherapy	0	39		1	24		1	24	
**Chemotherapy schedule**									
Inpatient	2	53	0.71	2	37	0.63	5	34	1.0
Outpatient	5	81		2	62		9	55	
**Chemotherapy protocol**									
Single agent	4	79	1.0	1	57	0.64	8	52	1.0
Multi agent	3	55		3	42		6	37	
**COVID-19 symptoms**									
Negative	6	142	0.09	5	101	1.0	14	92	0.14
Positive	1	1		0	1		1	0	

Significant values are in [bold].

### RT-PCR testing

Of 150 cancer patients, 7 (4.7%) cases had positive RT-PCR results. The average age for negative and positive groups was 53.3 and 58.2 years respectively, conferring a statistical difference between the two groups (*P*-value = 0.01). Of patients with negative test results, 57.1% (4/7) were female and 42.9% (3/7) were male. Moreover, of all patients who received negative test results, 61.5% were female and 38.5% were male. Five out of seven patients (71.4%) with positive results suffered from at least one comorbidity other than cancer (e.g., pulmonary, cardiovascular, chronic kidney disease (CKD), diabetes mellitus (DM), ischemic heart disease (IHD), hypertension (HTN), and liver diseases). The corresponding rate for patients who tested negative for COVID-19 was 25.8% (37 out of 143), conferring a statistical significance between the two groups (*P*-value = 0.02). Among patients with negative test results, 56.4% (79/140) had stage III or IV cancer and 43.6% (61/140) were diagnosed with stage I or II. These percentages were respectively 66.7% (4/6) and 33.3% (2/6) for patients who had positive findings for COVID-19 on RT-PCR. All of the positively tested patients^7^ had received chemotherapy as their treatment; four patients were being actively treated and the others were in the follow-up period. Moreover, 5 patients received chemotherapy in an outpatient setting, whereas, 2 were admitted to the hospital for the chemotherapy cancer treatment. Finally, among patients with positive COVID-19 test results, there were no significant differences between cases undergoing single-agent and multi-agent chemotherapy regimens.

The total number of cancer patients who tested positive for COVID-19 RT-PCR was 7 in our study. Table 2 demonstrates the clinical features, demographic characteristics, and details of cancer treatment. While none of these RT-PCR positive patients had positive IgM test results, 28.6% (2/7) of them tested positive for IgG. Interestingly, only one of our RT-PCR-positive patients was symptomatic, experiencing chill with no other associated symptoms of the COVID-19 disease, and the other patients were asymptomatic. Three individuals (42.9%) were diagnosed with stage IV cancer, and 28.6% had stage II cancer. The most frequent coexisting chronic disease was DM, which accounted for 57% of the comorbid conditions among the patients with positive COVID-19 tests. According to the P-values obtained from statistical analysis, comorbidity (P-value: 0.02) and older age (P-value: 0.01) were the only conditions that considerably affected the positivity rate of cancer patients.

**Table 2:** Clinical characteristics of COVID-19 infected cancer patients according to the RT-PCR results.

Case number	Age	IgG	IgM	Hx of COVID	Type of surgery	Symptoms	Tumor topography	Cancer stage	Comorbidity	Chemotherapy regimen	Number of cycles
2,619,623,235	58	Neg	Neg	Neg	TAH_BSO	None	Invasive endometrial carcinoma	III	DM	Paclitaxel + Carboplatin	–
475,346	54	Pos	Neg	Neg	–	Chill	AML	–	DM	Doxorubicin + Vincristine + Mesna	7
353,275	65	Neg	Neg	Neg	Hemicolectomy	None	Colon adenocarcinoma	II	None	5FU/LV	12
428,590	60	Pos	Neg	Neg	Partial gastrectomy	None	Gastric Adenocarcinoma	II	cerebrovascular disease	FOLFOX Capecitabine	7
875,409	54	Neg	Neg	Neg	–	None	Metastatic invasive ductal carcinoma of breast	IV	DM HTN	Capecitabine	4
427,862	69	–	–	Neg	Rectal proctectomy	None	Adenocarcinoma of Rectum	IV	None	FOLFOX	9
388,754	58	–	–	Neg	–	None	Metastatic leiomyosarcoma	IV	DM	Ifosfamide + Mesna + Doxorubicin	3

### Antibody testing

#### IgG serology test

Another serology-based test employed was IgG antibody serology for detecting history of COVID-19 infection. Average age for the positive and negative groups were 55 and 52.7 years respectively. The prevalence of positive COVID-19 IgG test of the cancer patients was 14.02%. Fifteen cancer patients tested positive for COVID-19 infection, eleven of them were female (73.3%). About 53.3% of the patients with positive test results had at least one comorbid condition. Among the positive group, 80% were in the middle of their course of treatment (chemotherapy, radiotherapy or both) and the other 20% were in their follow-up period. About 50% of the patients whose test results came back positive had stage I or II cancer, and the other half were diagnosed with stage III or IV. Overall, thirteen out of fourteen (92.8%) patients had received chemotherapy as a part of their treatment (the details of the treatment can be seen in Table 3), while one underwent radiation therapy (7.1%). Among the cases who tested positive and received chemotherapy as their cancer treatment, the protocol of 42.9% (6/14) consisted of multi-agent regimens. Moreover, the chemotherapy setting was outpatient in 9 out of 14 patients, and inpatient for others.

**Table 3:** Clinical characteristics of cancer patients with history of COVID-19 infection according to IgG serology results.

Case number	Age	RT-PCR	History of COVID	IgM	Type of surgery	Symptoms	Tumor topography	Cancer stage	Comorbidity	Chemotherapy regimen	Number of cycles
251,207	60	Neg	Neg	Neg	–	–	Recurrence of Rectal Adenocarcinoma	IV	DM-HTN	Capecitabine	1
497,260	54	Neg	Neg	Neg	–	–	Colon adenocarcinoma	II	DM-HTN	FOLFOX	6
500,536	67	Neg	Neg	Neg	BCS + ALND	–	Invasive ductal carcinoma of breast	II	–	AC	2
465,545	57	Neg	Pos	Neg	TAH + BSO	–	Endometrial adenocarcinoma	III	DM	Paclitaxel + Carboplatin	2
502,145	53	Neg	Neg	Neg	Total colectomy	–	Adenocarcinoma of colon	II	–	FOLFOX	3
475,346	54	Pos	Neg	Neg	–	Chill	AML	–	DM	Doxorubicin + Vincristine + Mesna	7
504,336	44	Neg	Neg	Neg	–	–	Metastatic breast cancer	IV	–	Gemcitabine + Vinorelbine	2
476,317	51	Neg	Neg	Neg	TAH + BSO	–	Serous Adenocarcinoma of ovary	III	–	Paclitaxel + Carboplatin and Doxorubicin + Carboplatin	22
237,432	69	Neg	Neg	Neg	–	–	Prostatic adenocarcinoma	II	HTN	–	–
289,523	51	Neg	Neg	Neg	MRM	–	Invasive ductal carcinoma of breast	II	–	AC Docetaxel + Carboplatin	10
301,947	44	Neg	Neg	Neg	–	–	Metastatic Breast Cancer	IV	–	Capecitabine + Oxaliplatin	2
428,590	60	Pos	Neg	Neg	Partial gastrectomy	–	Gastric Adenocarcinoma	II	–	FOLFOX Capecitabine	7
486,829	61	Neg	Neg	Pos	–	–	SCC of Esophagus	III	HTN-IHD	5FU + Paclitaxel + Cisplatin	7
497,251	63	Neg	Neg	Pos	Rt hemicolectomy	–	Adenocarcinoma of Colon	II	DM-HTN-IHD	FOLFOX	5
295,783	39	Neg	Neg	Pos	–	–	Metastatic SCC of larynx	IV	–	Gemcitabine	3

According to the data, fifteen patients tested positive for a history of COVID-19 infection based on IgG test results, whose demographic and clinical characteristics are summarized in Table 3. Among these fifteen cases, only 13.3% (2/15) and 20% (3/15) had positive RT-PCR and IgM test results, respectively. Only one patient had experienced chill and his/her infection was symptomatic. Interestingly, both of the RT-PCR and IgG test results of this case came back positive. Meanwhile, one out of fifteen patients who tested positive had a history of COVID-19. Seven out of fifteen patients had at least one of the comorbid conditions, the most prevalent of them were DM and HTN. Of the patients with positive tests, 46.7% were diagnosed with stage II cancer. Three individuals were admitted to the inpatient setting, while the others participated in the outpatient program.

#### IgM serology test

IgM serologic-based testing of COVID-19 was performed as well, the result of which can be seen in Table 4. The rate of IgM positivity was about 4.7% (5/107). The average age of the populations with positive and negative test results were respectively 54.3 and 51.3 years. Three out of these five patients (60%) were female. Moreover, 40% of the population with positive test results were patients with at least one comorbidity. Regarding the cancer journey state in patients with positive IgM serology, 3 (60%) were in the middle of their treatment for cancer (chemotherapy, radiotherapy or both) and 2 (40%) were being routinely followed after completion of their cancer treatment. Three out of five patients had stage I or II cancer and the others were diagnosed with stage III or IV. Among the patients whose IgM test results came back positive, 80% received chemotherapy and the others were given radiotherapy as their cancer treatment program.

**Table 4:** Clinical characteristics of COVID-19 infected cancer patients according to the IgM test results.

Case number	Age	RT-PCR	History of COVID	IgG	Type of surgery	Symptoms	Tumor topography	Cancer stage	Comorbidity	Chemotherapy regimen	Number of cycles
508,842	53	Neg	Neg	Neg	–	–	Lung Adenocarcinoma	II	–	–	–
497,251	63	Neg	Neg	Pos	Rt hemicolectomy	–	Adenocarcinoma of Colon	II	DM-HTN-IHD	FOLFOX	5
295,783	39	Neg	Neg	Pos	–	–	Metastatic SCC of larynx	IV	–	Gemcitabine	4
486,829	61	Neg	Neg	Pos	–	–	SCC of Esophagus	III	HTN-IHD	5FU + Paclitaxel + Cisplatin	7
419,898	39	Neg	Neg	Neg	BCS + ALND	–	Invasive ductal carcinoma of breast	II	–	AC Docetaxel	7

The results of IgM serology tests showed that five patients were positive for COVID-19 infection. Among these five patients, three cases were also positive for IgG serology tests and all were negative when being tested by RT-PCR. Meanwhile, none of these individuals had a history of COVID-19 infection, and while being positive, they did not develop any symptoms. Three out of five patients have stage II cancer and the others’ pathological stages were stage III and IV. Half of the patients with positive results were admitted to the inpatient setting and the others had outpatient service.

### Comparison with control group

Comparison results between cancer patients and control group is presented in Table 5 Out of 129 participants in the anti-body control group, 11 (8.5%) had a positive IgM test result and 18 (14.0%) had positive IgG test result. No significant difference was observed between cancer and non-cancer patients regarding COVID-19 anti-body tests. A level of association was detected between patient type and molecular test results. In the molecular test control group, 125 (13.6%) had positive RT-PCR results which was significant compared to 7 (4.6%) cancer patients with positive RT-PCR results. We obtained age adjusted odds ratio for positive RT-PCR outcome using a logistic regression which revealed OR = 0.33 (CI: 0.15–0.73), meaning that cancer patients were 3 times less likely to have a positive molecular test result.

**Table 5:** Statistical comparison of molecular and anti-body test results in cancer and non-cancer patients.

	RT-PCR result			IgM serology result			IgG serology result		
	Pos	Neg	*P*-value	Pos	Neg	*P*-value	Pos	Neg	*P*-value
**Patient type**									
Cancer	7	143	**0.003**	5	102	0.36	15	92	0.86
Non-cancer	125	795		11	118		18	111	

Significant values are in [bold].

## Discussion

Since the beginning of the COVID-19 pandemic, a growing body of research has been investigating the impact of various factors such as comorbidity on the severity of the complications that arise from this viral infection^12–14^. It is suggested that cancer might put patients, who have higher levels of inflammatory and coagulopathy markers, at higher risk of severe COVID-19 infection and mortality^15^. In addition, since tumor growth and cancer treatments suppress their immune system, they might be at even higher risks^16^. On the other hand, regular hospital visits increase their interaction with infected patients^11^, and as a result, the percentage of coronavirus tests that come back positive is expected to be higher in the cancer population compared to the public, urging physicians to modify their strategies for cancer treatment^17^. Performing routine COVID-19 testing prior to the cancer treatment is highly recommended by various studies, as the clinical course of COVID-19 can range from asymptomatic or mild to severe disease. While a delay in cancer treatment may result in poor cancer control and unfavorable survival outcomes, there is a possibility that cancer treatment can predispose patients to higher risk for severe COVID-19^18^. Meanwhile, little information exists regarding COVID-19’s effect on treatment course and risk of infection as a result of continued therapy^19^. In this report, we gathered data from 150 cancer patients of Imam Hossein Hospital and our main goal was to determine whether cancer patients are different from general population regarding the positivity rate of various COVID-19 tests. Interestingly, the treatment modality, chemotherapy schedule or regimen, or the stage of the disease had no impact on the positivity rate of COVID-19 RT-PCR or the serological tests. For example, all of the seven patients had received at least one type of cancer treatment which was chemotherapy with or without surgery, therefore, it is unlikely that COVID-19 test positivity have any significant association with the type of treatment modality. Regarding other variables assessed in our study, having other comorbidities and older age made significant statistical differences in our RT-PCR positive group, as 70% of the patients suffered from at least one comorbid condition.

Elevated IgG serum levels in two patients (4.2 and 9.8) could mean that they have had prior exposure to COVID-19 infection and their RT-PCR had not turned negative yet. Moreover, since these 2 patients did not report any history of COVID-19 disease, they were probably asymptomatically infected. Our results showed no significant difference in the positivity rate of IgG and IgM test results between cancer patients and non-cancer patients. Significant negative association was observed between having cancer and positive RT-PCR test. Individuals in the control group were 3 times more likely to have a positive RT-PCR test (13.6% Vs. 4%). The positive test rate in non-cancer patients, however, matches closely with the reported percentage of positive tests among the general population in the same period. In Iran, the percentage of positive COVID-19 tests among the general population was about 12.6% on Oct 1st, 2020^20^. This may suggest that since cancer patients consider themselves more vulnerable to environmental risk factors, they follow self-isolation and social distancing recommendations more seriously.

Our results contribute to the idea that routine baseline testing prior to or during anti-cancer therapy may not be as essential as it is recommended in some studies^18^. This conclusion stems from the fact that, according to our results, having COVID-19 related symptoms or being asymptomatic made no significant difference in COVID-19 test positivity rate. In addition, the rate of RT-PCR positivity was significantly lower than the general population. As a result, the same rule of not conducting routine COVID-19 testing in the general population applies for cancer patients and we even have more evidence supporting this practice in cancer patients. Therefore, common screening methods such as asking about the symptoms of COVID-19 disease in each medical visit of cancer patients (either for treatment or as follow-up) is sufficient.

The higher mortality rate of cancer patients affected with COVID-19 compared to the general population^5^ may suggest considering routine COVID-19 testing in a subgroup of cancer patients (not all of them). One study suggests considering some criteria including socioeconomic status, accessibility of the testing resources, the capacity of the laboratory, and regional prevalence when trying to determine the necessity of the tests^18^. In our study, having comorbidities and older age were associated with a higher probability of positive RT-PCR test. This suggests that these groups may benefit most from routine COVID-19 testing. One of the limitations of our study was the smaller sample size of the cancer patients compared to non-cancer cohort which represents more diverse demographic data. Also, we were unable to conduct a multivariate analysis due to the small sample size. Therefore, conducting a similar study with a larger sample size and multivariate analysis could identify a subgroup of cancer patients undergoing active anti-cancer treatment who may be considered for routine COVID-19 testing.

## Conclusion

Although we could conclude that the probability of cancer patients having a positive routine test is less than non-cancer patients, and the concept that all cancer patients should be routinely tested for COVID-19 is probably not right, there may be a subset of cancer patients who may benefit from routine testing (patients with comorbidities or older age). However, the effect of factors including receiving or not receiving cancer treatment or the impact of various cancer treatment modalities cannot be addressed due to our limited sample size. Importantly, these results could not be studied in a multivariate model.

## Data Availability

The datasets generated during and/or analyzed during the current study are available from the corresponding author on reasonable request.
